# Unilateral biportal endoscopic lumbar hemivertebra resection with bilateral percutaneous fixation in a pediatric patient: a case report

**DOI:** 10.3389/fped.2026.1773395

**Published:** 2026-03-18

**Authors:** Peng Zhang, Zijie Sun, Kun Li, Kefeng Xu, Genyang Jin, Hengyan Zhuge, Chengyun Wang, Qi Liu

**Affiliations:** 1 Department of Orthopedics, The 904th Hospital of Joint Logistic Support Force of PLA, Wuxi, China; 2 Department of Pediatric Orthopedics, The First Affiliated Hospital of Xiamen University, Xiamen, China

**Keywords:** congenital scoliosis, hemivertebra resection, minimally invasive surgery, pediatric spine, percutaneous pedicle screw, unilateral biportal endoscopy

## Abstract

**Background:**

Minimally invasive techniques are gaining interest in pediatric spinal deformity surgery. Unilateral biportal endoscopy (UBE) offers precise visualization with minimal tissue disruption. However, achieving sufficient biomechanical stability after a major osteotomy, such as hemivertebra (HV) resection, remains critical.

**Methods:**

A 7-year-old girl with progressive congenital scoliosis (CS) (segmental Cobb angle 32°) due to a left L4/L5 semi-segmented HV underwent complete HV resection via UBE. Using a fully percutaneous technique, the UBE portals were reused for ipsilateral pedicle screw insertion at L4 and L5. Two additional contralateral stab incisions enabled bilateral percutaneous screw placement, followed by rod insertion and compression.

**Results:**

The 210-minute procedure achieved a 56% segmental correction (32° to 14°), with estimated blood loss of 100 mL. At 6-month follow-up, correction was maintained (Cobb 14°) with evidence of radiographic fusion and no complications. The entire procedure required only four stab incisions (each 1–1.5 cm).

**Conclusion:**

UBE enables precise lumbar HV resection under direct visualization. When synergized with bilateral percutaneous short-segment fixation, it provides the mechanical integrity essential for maintaining correction. This combined protocol successfully marries minimal tissue disruption with surgical efficacy, offering a promising alternative within the minimally invasive armamentarium for pediatric spinal deformity.

## 1 Introduction

Posterior hemivertebra (HV) resection with short-segment fusion represents the standard treatment for progressive congenital scoliosis (CS) caused by HV (1–3). Although effective, the conventional open approach requires extensive paraspinal muscle dissection, which can lead to postoperative pain, muscle atrophy, and longer recovery in children. Unilateral biportal endoscopy (UBE) surgery has emerged as a minimally invasive alternative, providing magnified visualization through separate working and viewing channels (4–6). However, its application in pediatric lumbar HV resection remains relatively unexplored. A prior report on thoracic HV resection using UBE with unilateral percutaneous fixation demonstrated technical feasibility but resulted in implant failure due to insufficient stability (7). This experience underscores a critical principle: the pursuit of minimal invasiveness must not compromise the mechanical demands of spinal deformity correction. Building upon this insight, we herein present—to our knowledge—the first reported case of L4/L5 lumbar HV resection using UBE combined with bilateral percutaneous pedicle screw fixation in a pediatric patient. This report aims to describe the technical feasibility, emphasize the achieved stability, and present early outcomes of this integrated approach, which seeks to harmonize minimal access with robust biomechanical support.

## 2 Case presentation and surgical technique

**Figure 1:**
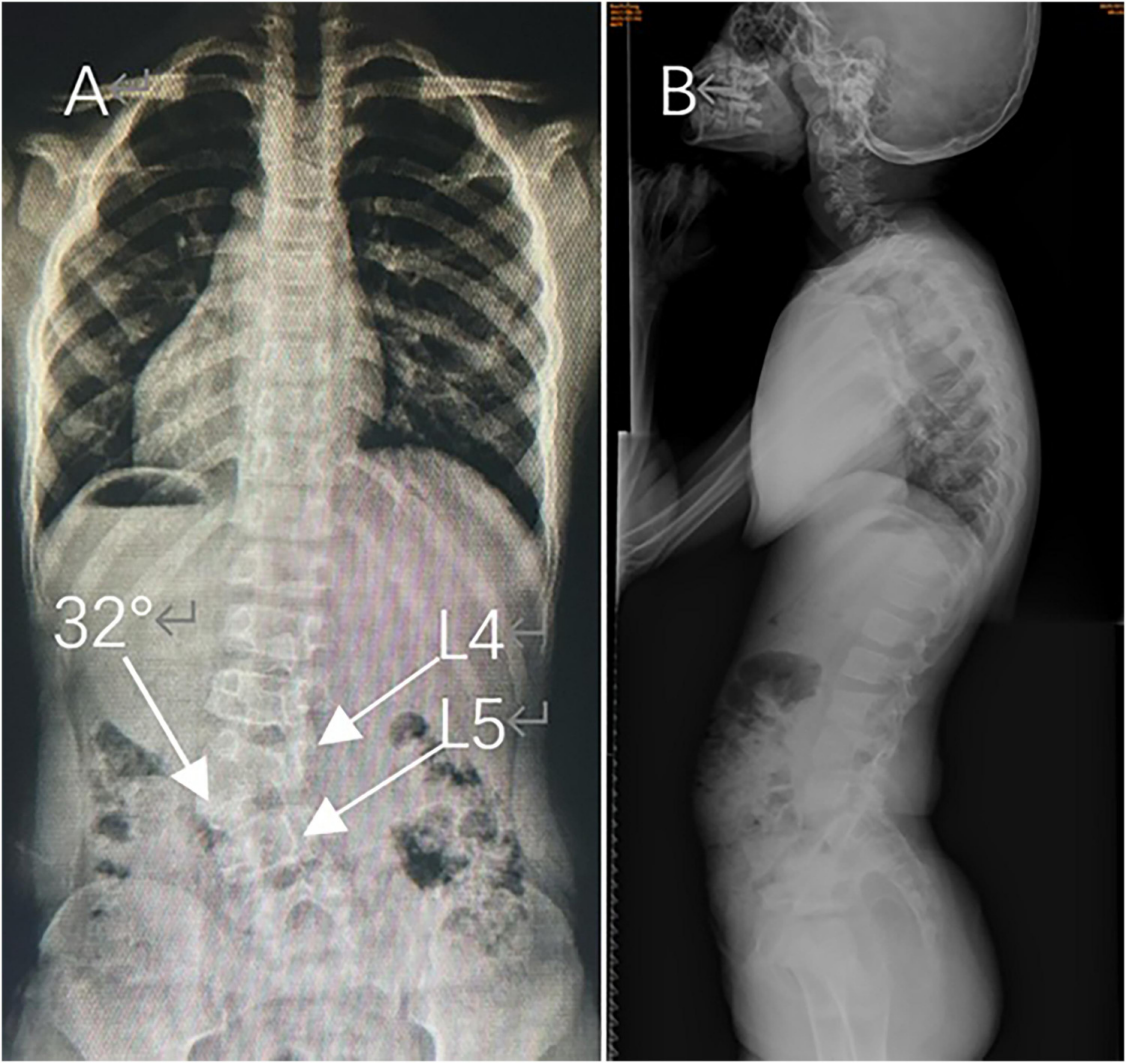
Preoperative full-spine standing radiographs. **(A)** Anteroposterior (AP) view demonstrating CS with a left L4/L5 semi-segmented HV (white arrow), a segmental Cobb angle of 32°, and compensatory sacral obliquity. (Levels L4 and L5 are labeled.) **(B)** Lateral view showing preserved physiological lumbar lordosis.

**Figure 2:**
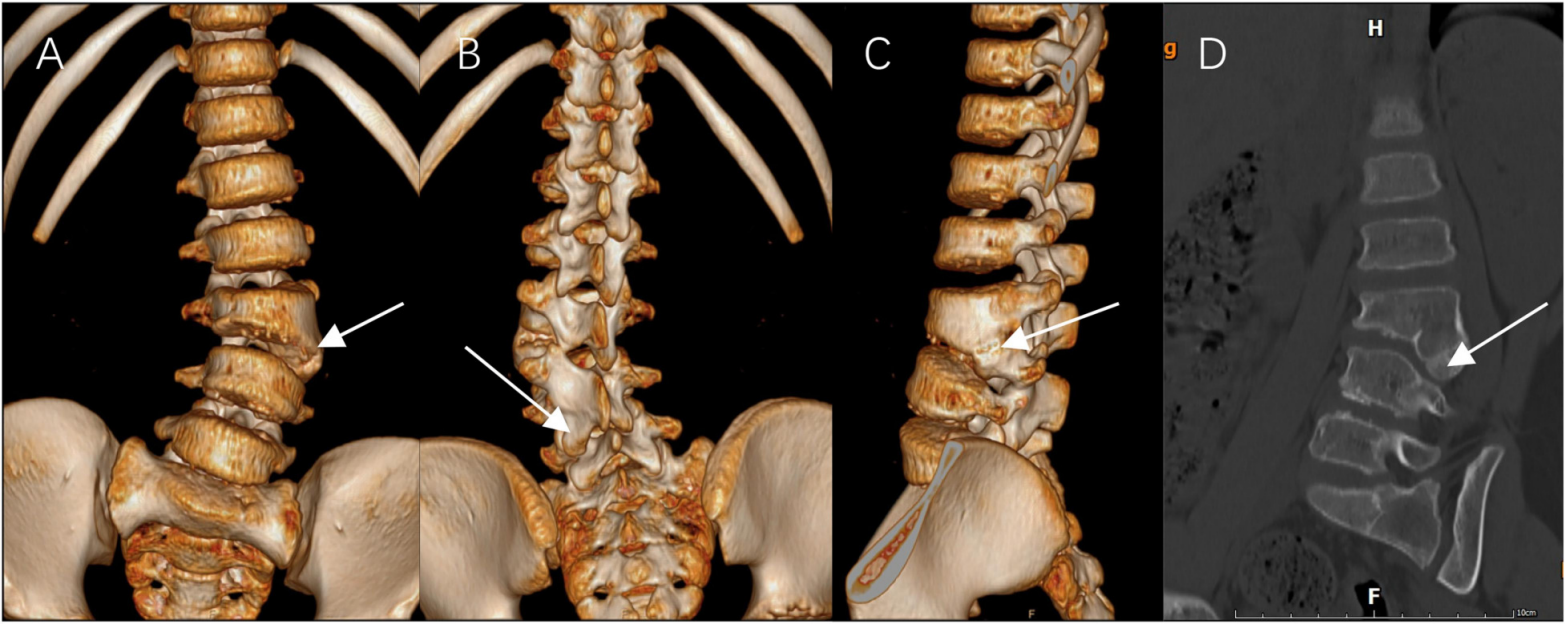
Preoperative 3D CT reconstructions of the L3/L4 HV (white arrow). **(A)** Anterior view. **(B)** Posterior view. **(C)** Left lateral view. **(D)** Sagittal cut view.

A 7-year-old girl presented with a noticeable left lumbar prominence and occasional self-correcting trunk shift. Neurological examination revealed no motor, sensory, or reflex deficits. Radiographs confirmed a left L4/L5 semi-segmented HV with a segmental Cobb angle of 32° and compensatory sacral obliquity (Figure 1). (Full-spine radiographs confirmed the presence of 12 thoracic vertebrae and lumbarization of S1, resulting in an additional lumbar segment.) 3-dimensional CT revealed a typical semi-segmented HV (Figure 2). Preoperative MRI showed no intraspinal anomalies, and renal/cardiac ultrasound screenings were normal. Given documented curve progression despite one year of brace treatment, surgical intervention was indicated.

**Figure 3:**
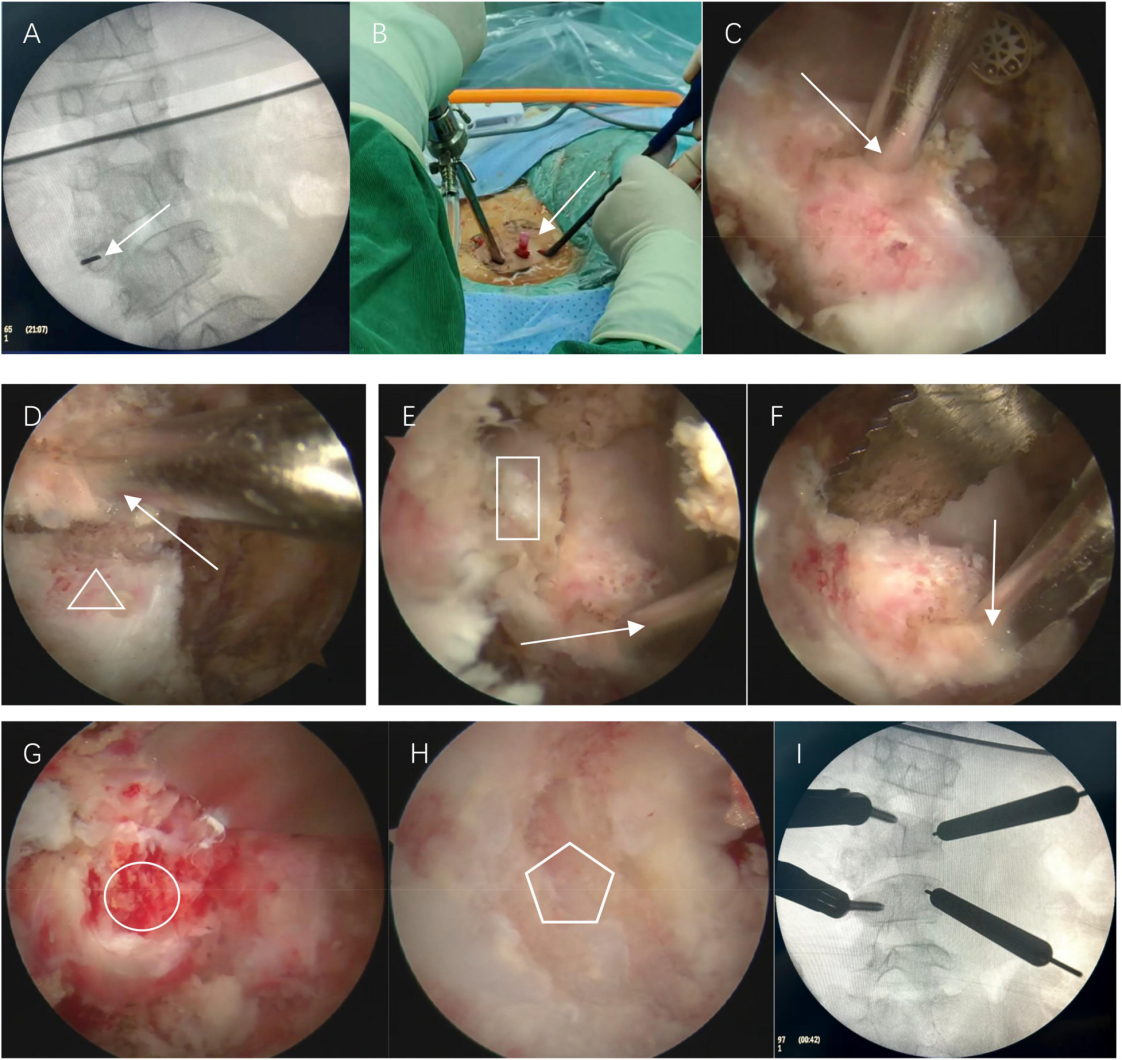
Intraoperative views: **(A)** fluoroscopic image confirming the percutaneous placement of a needle (arrow) into the pedicle of the left L3/L4 HV. **(B)** Intraoperative photographs showing endoscopic viewing and working portals were established. **(C)** Endoscopic view showing the workspace. **(D)** Endoscopic view showing the resection of the transverse process (triangle). **(E)** Endoscopic view showing the resection of the superior articular process (rectangle). **(F)** Endoscopic view showing the resection of HV lamina with ultrasonic bone scalpel. **(G)** Endoscopic view showing the pedicle(circle) after the resection of transverse process, inferior articular process and lamina. **(H)** Endoscopic view showing the bleeding lower endplate of L3(pentagon) following complete removal of the HV. **(I)** Fluoroscopic image confirming the accurate placement of the Polyaxial percutaneous pedicle screws.

The patient was positioned prone under general anesthesia, with continuous SEP/MEP monitoring. Fluoroscopy-guided localization of the L4/L5 level was performed. A percutaneous needle was inserted into the left L4/L5 HV pedicle as a landmark. Endoscopic viewing and working portals were established 1.5 cm from the midline, approximately 1 cm cranial and caudal to the target pedicle. The coagulation of soft tissues was achieved using a radiofrequency ablator (ARS600, Bangshi Medical; Ablate 7: 220W; Coag 3: 23W). Irrigation was performed with normal saline via a completely open system (bag height 1.8 m). Under endoscopic visualization, the transverse process, superior and inferior articular process, and HV lamina were resected using an ultrasonic bone scalpel. After identifying and protecting the traversing nerve roots along the medial pedicle wall with minimal retraction, the pedicle was drilled and the HV was meticulously segmented using a bone chisel and ultrasonic bone cutter. After obtaining adequate bone blocks for grafting, a burr was used to excise the residual wall. Following this, the adjacent discs were carefully excised down to the bleeding endplates (Figure 3). During this phase, hemostasis and safe retraction of the traversing nerve roots under endoscopic visualization were given paramount attention.

**Figure 4:**
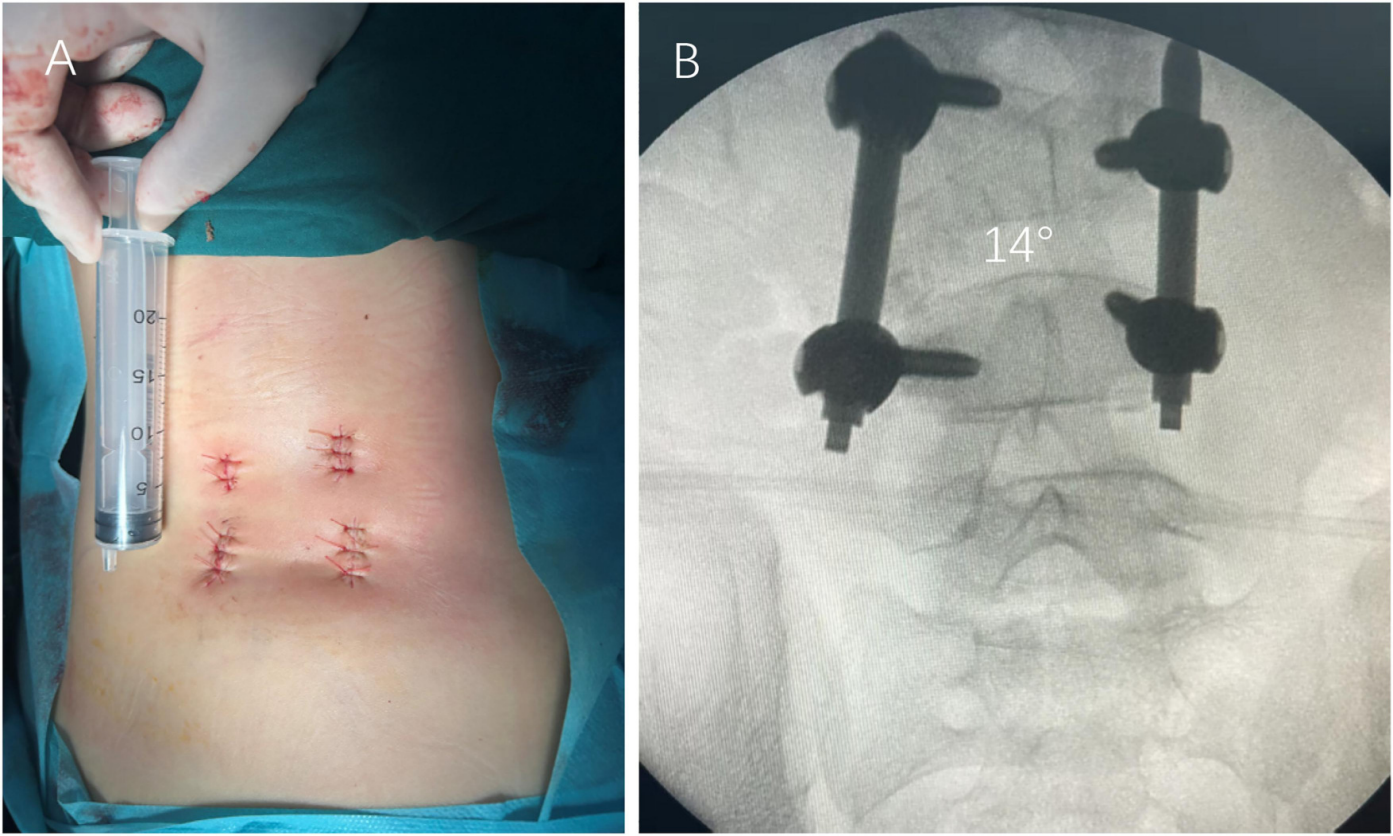
Minimally invasive incisions and immediate postoperative fixation. **(A)** Postoperative photograph of the lower back, showing four small incision sites. **(B)** Intraoperative fluoroscopic AP view after instrumentation, demonstrating bilateral percutaneous pedicle screws at L4 and L5, a pre-contoured rod, and closure of the resection gap.

Subsequently, the UBE working and viewing portals were reused as percutaneous entry points for guidewire insertion into the left L4 and L5 pedicles under fluoroscopic guidance. Two additional symmetrical stab incisions (1.5 cm each) were made on the right side for contralateral L4 and L5 guidewire placement. Polyaxial percutaneous pedicle screws (5.0 mm diameter, 35 mm length) were inserted bilaterally based on preoperative CT measurements (pedicle diameter 4.5–5.0 mm, trajectory length 35–40 mm). Throughout the procedure, fluoroscopy was used only for key steps—localizing the L4/L5 level and confirming screw placement—to minimize radiation exposure. Pre-contoured rods were passed subfascially, and gradual compression was applied to close the osteotomy gap. Autologous bone graft obtained from the resected HV was then packed into the residual defect for interbody fusion. Prior to graft placement, hemostasis was meticulously achieved. All four small incisions were closed without drain placement. Total operative time was 210 min with an estimated blood loss of 100 mL (Figure 4).

## 3 Results

**Figure 5:**
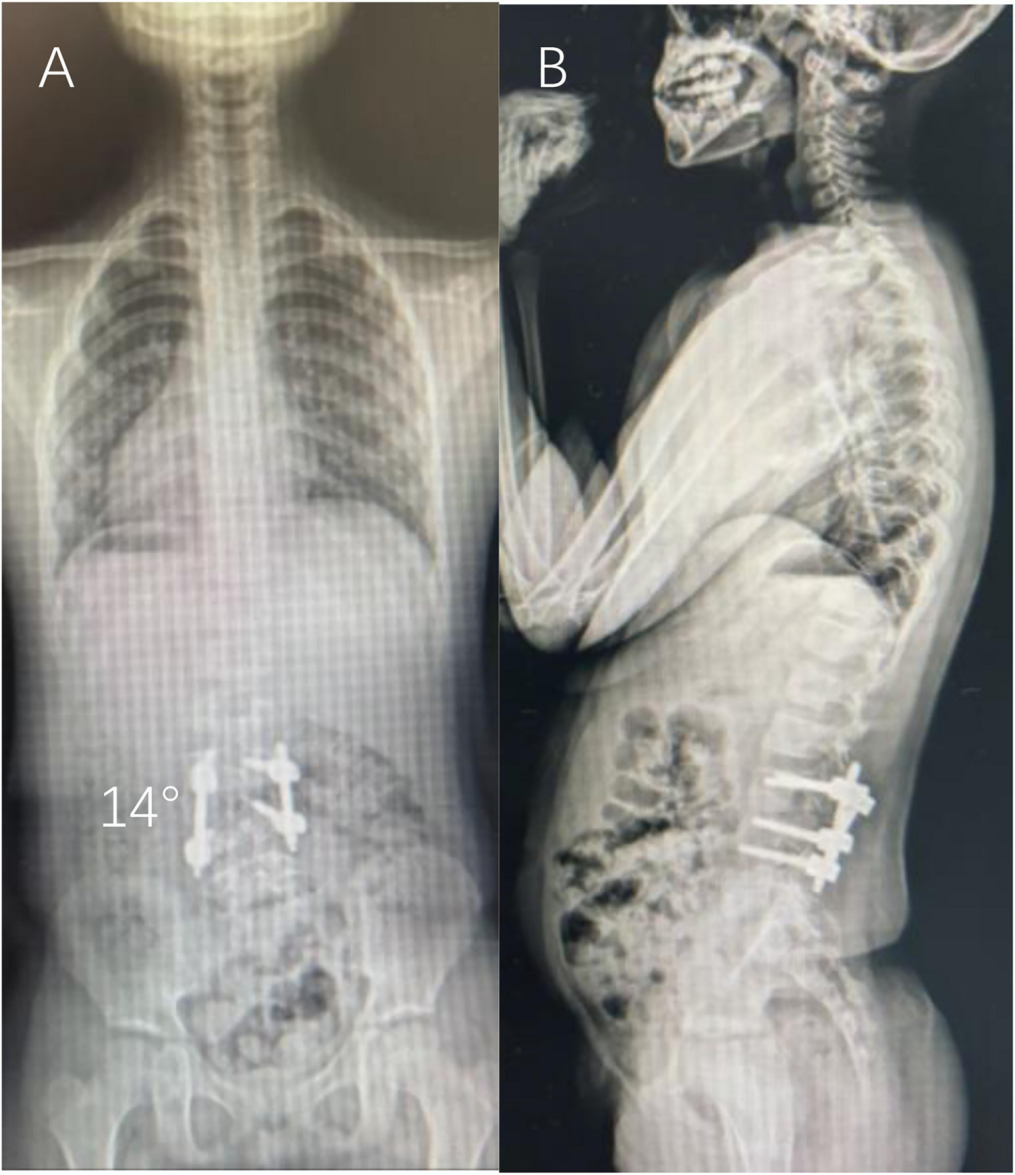
Postoperative full-spine standing radiographs at 6-month follow-up. **(A)** AP view showing maintained coronal correction with a Cobb angle of 14° and evidence of bony fusion on radiographs at the L4–L5 level. **(B)** Lateral view confirming the preservation of physiological lumbar lordosis.

The patient was mobilized on postoperative day 2 wearing a custom thoracic-lumbar-sacral orthosis (TLSO). The postoperative day 2 VAS pain score was 5. The hospital stay was 12 days. Immediate postoperative radiographs demonstrated correction of the segmental Cobb angle to 14° (Figure 4). At the 6-month follow-up, the correction was maintained at 14° with no radiographic signs of implant loosening, failure, or loss of fixation, suggesting early radiographic fusion. Definitive assessment of solid fusion is planned with CT at 1 year postoperatively (Figure 5). Neurological status remained intact throughout, no cerebrospinal fluid leak, surgical site infection, or reoperation occurred. The patient and her family were satisfied with the postoperative appearance and functional recovery. The cosmetic outcome was excellent, with only four minimal scars. The complete timeline from diagnosis to follow-up is summarized in Table 1.

**Table 1:** Timeline of management and follow-up.

Timepoint	Event
June 2023	Initial diagnosis of CS
June 2023 – June 2024	Observation, noted progression
June 2024 – June 2025	Brace treatment, cosmetic improvement but angular progression
June 2025	Surgical decision
(Surgery Date)	UBE-assisted HV resection with bilateral percutaneous fixation
Postoperative day 2	Mobilization begun, VAS pain score 5
Hospital stay	12 days
Bracing period	3 months
Follow-up	Monthly for first 3 months, then every 3 months thereafter until adulthood
6-month follow-up	Radiographic and clinical assessment

## 4 Discussion

The surgical approach for HV has evolved significantly. Traditional combined anterior-posterior resection has been largely superseded by single-stage posterior-only approaches. Enhanced by advanced pedicle screw systems, these techniques effectively transmit corrective forces across the anterior and middle spinal columns, achieving substantial deformity correction (54%–77% scoliosis reduction) with reduced invasiveness and preserved growth potential (2, 8, 9). However, conventional posterior “blind” resection, reliant on fluoroscopy and tactile feel, carries inherent risks during manipulation of the HV's medial and posterior walls, including dural or venous plexus injury, incomplete fragment removal, and potential iatrogenic damage to ventral great vessels.

The UBE technique, initially developed for lumbar degenerative disease, has expanded to address various spinal pathologies (4, 10–13). Its application in HV resection represents a logical step in this minimally invasive evolution. Unlike open posterior approaches requiring extensive subperiosteal dissection, UBE utilizes natural intermuscular planes to establish working corridors, significantly reducing paraspinal muscle trauma and blood loss. Crucially, its dual-portal design provides continuous, magnified endoscopic visualization coupled with the flexibility of using conventional open-surgery instruments. This is essential for precise, real-time osseous manipulation and direct protection of neural elements and adjacent tissues during HV resection, effectively mitigating the risks associated with “blind” maneuvers. Direct visualization also facilitates confirmation of complete HV removal and thorough endplate preparation, which are critical for successful fusion.

This report details a successful technical synthesis that builds upon this evolving landscape. We integrated the precise, minimally invasive resection capability of UBE with the robust stability of bilateral segmental instrumentation. This approach directly addresses a critical gap identified in our prior experience, where UBE-assisted thoracic HV resection with unilateral percutaneous fixation failed due to insufficient stability (7). By evolving to a fully percutaneous bilateral fixation strategy—repurposing the UBE portals for ipsilateral screws and adding only two contralateral stab incisions—we achieved a stable four-screw, two-rod construct. This eliminates open midline dissection, resulting in a remarkably small surgical footprint of four 1–1.5 cm incisions, while providing the mechanical integrity essential for maintaining correction after a major osteotomy. Biomechanically, a unilateral construct creates a cantilever effect on the resection side, while bilateral segmental fixation establishes a stable load-sharing ring, which is crucial for an osteotomy that disrupts all three spinal columns.

Several technical aspects specific to the pediatric lumbar spine warrant emphasis. First, while the absence of rib heads facilitates lumbar access compared to the thoracic spine, it is counterbalanced by the need for meticulous endoscopic management of the larger and more mobile lumbar nerve roots, demanding precise osteotomy and judicious retraction. The superb visualization of UBE is particularly advantageous here. Second, the restrictive dimensions of pediatric lumbar pedicles present a challenge. The use of appropriately sized 5.0 mm diameter percutaneous screws was based on preoperative CT measurements and was crucial to achieve adequate bony purchase while mitigating the risk of pedicle breach—a common concern in pediatric instrumentation (14–16). The choice of short-segment fusion is justified in this isolated HV case, as it directly targets the pathological segment, preserves motion segments, and, when combined with a stable bilateral construct, provides sufficient load-bearing capacity for fusion.

The favorable outcome reinforces a fundamental principle: in pediatric spinal deformity correction, the pursuit of minimal invasiveness must not compromise mechanical stability. The previous failure of unilateral fixation (7) serves as a cautionary tale, highlighting the high asymmetric and rotational forces across a resection gap. Bilateral segmental fixation, even over a short fusion, provides the necessary load-sharing and resistance to these forces during the critical healing period. This construct effectively bridges the osteotomy, controls the segment, and creates a favorable environment for fusion. Our experience suggests that for UBE-assisted major osteotomies, bilateral fixation may represent a more stable option and could be considered in similar cases.

The degree of HV segmentation primarily predicts progression risk; from a technical standpoint, endoscopic resection is feasible for fully, semi-, or non-segmented types. In non-segmented variants, identifying the resection margin may be more challenging, though a cartilaginous cleft between the HV and adjacent vertebra is usually discernible under endoscopic magnification and with fluoroscopic guidance. Age is a practical consideration: percutaneous screw placement becomes technically demanding in very young children, and we suggest an age threshold of approximately 5 years, similar to conventional approaches. Thoracic HV resection via UBE may be less favorable due to the frequent need for multi-level instrumentation (often ≥8 screws), prolonged operative time, and risk of significant pleural effusion if pleural injury occurs. While larger curve magnitudes or lumbosacral HV may increase technical difficulty, they are not absolute contraindications. Therefore, this approach is best applied by surgeons experienced in both advanced spinal endoscopy and pediatric deformity surgery, in carefully selected patients with progressive deformities amenable to short-segment correction.

This technique harmonizes several goals critical to pediatric care: reduced surgical trauma, which may translate to less postoperative pain and faster recovery; enhanced cosmesis with minimal scarring; and uncompromised correction. Looking forward, this protocol could be applied to other focal pediatric spinal pathologies requiring osteotomy and fusion. However, the potential learning curve associated with endoscopic osteotomy and percutaneous pediatric screw placement must be acknowledged.

This report is inherently limited by its nature as a single-case description with a 6-month follow-up. While early results are promising, longer-term follow-up is essential to confirm maintenance of correction, solid fusion into adulthood, and the absence of late complications such as adjacent segment degeneration. Further experience and comparative studies are needed to fully validate the efficacy, safety, and benefits of this technique in a larger pediatric population.

## 5 Conclusion

UBE-assisted lumbar HV resection combined with bilateral percutaneous short-segment fixation is a feasible and effective minimally invasive strategy for pediatric CS. It successfully balances the goals of reduced tissue trauma and superior visualization with the non-negotiable requirement for biomechanical stability. This bilateral percutaneous protocol represents a meaningful and necessary evolution in minimally invasive HV resection techniques, directly addressing the stability limitations of earlier unilateral approaches and presenting a promising and biomechanically sound alternative in the surgical armamentarium for pediatric spinal deformity.

## Data Availability

The original contributions presented in the study are included in the article/Supplementary Material, further inquiries can be directed to the corresponding author.
